# Supplementary material for: Controllability changes pain perception by increasing the precision of expectations
Source: Nat Commun. 2025 Nov 18;16:10113. doi: 10.1038/s41467-025-66038-7 (PMC12627469; doi:10.1038/s41467-025-66038-7)
Supplement: Supplementary file 1 — Supplementary Information [file 41467_2025_66038_MOESM1_ESM.pdf]

Supplementary Information

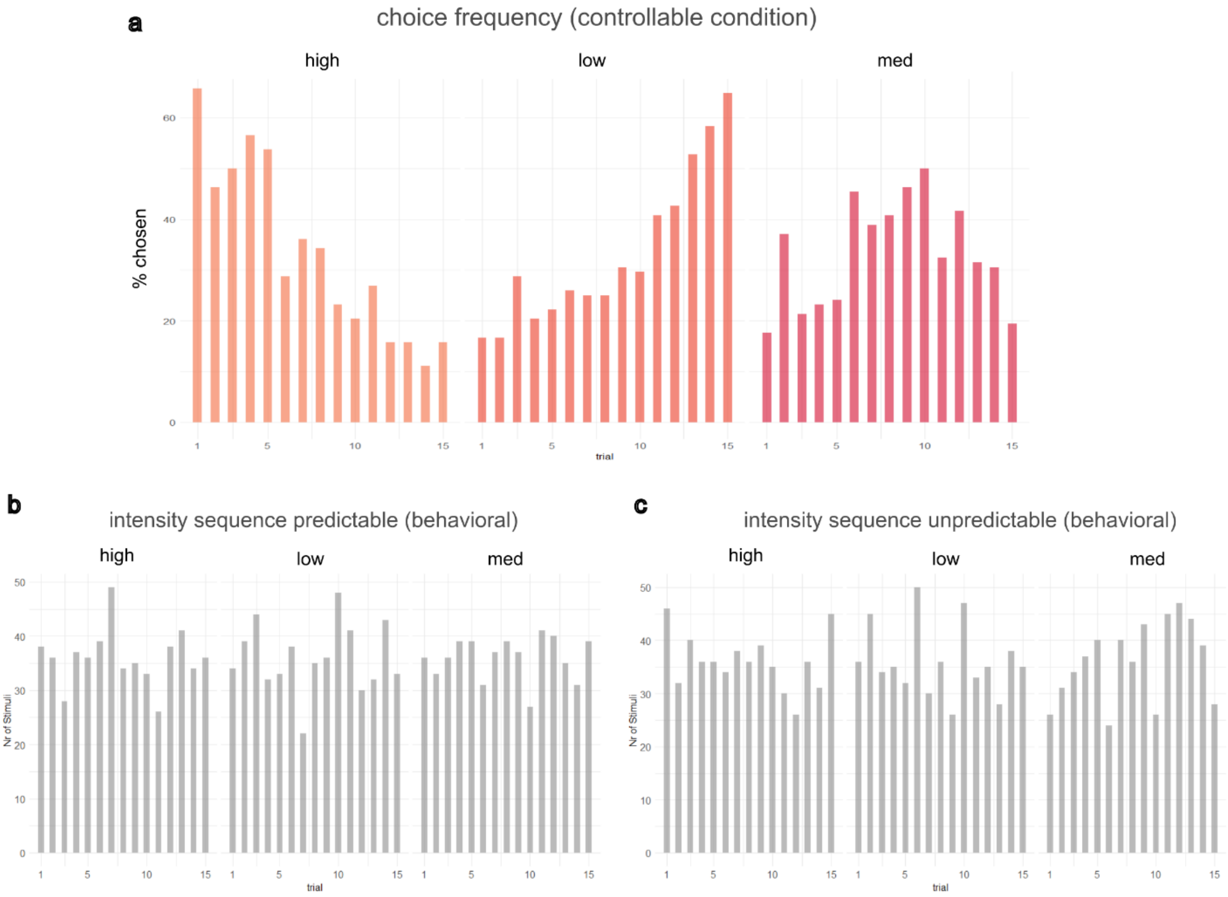

Figure S1 | Choice behavior and intensity sequences in the behavioral sample. (a) Participants showed negative discounting behavior during the controllable condition choosing high painful stimuli at the beginning and keeping low painful stimuli until the end of a run. A small divergence of the overall pattern can be observed in the first three trials, where an increase is visible for all intensity levels. This might possibly be related to participants exploring all three options before following a strategy for the rest of the run. **(b, c)** Because we had no prior information about this behavior before starting the data collection of the behavioral sample, intensity sequences in the unpredictable and predictable condition were created with uniform distributions. The conditions were not optimally matched.

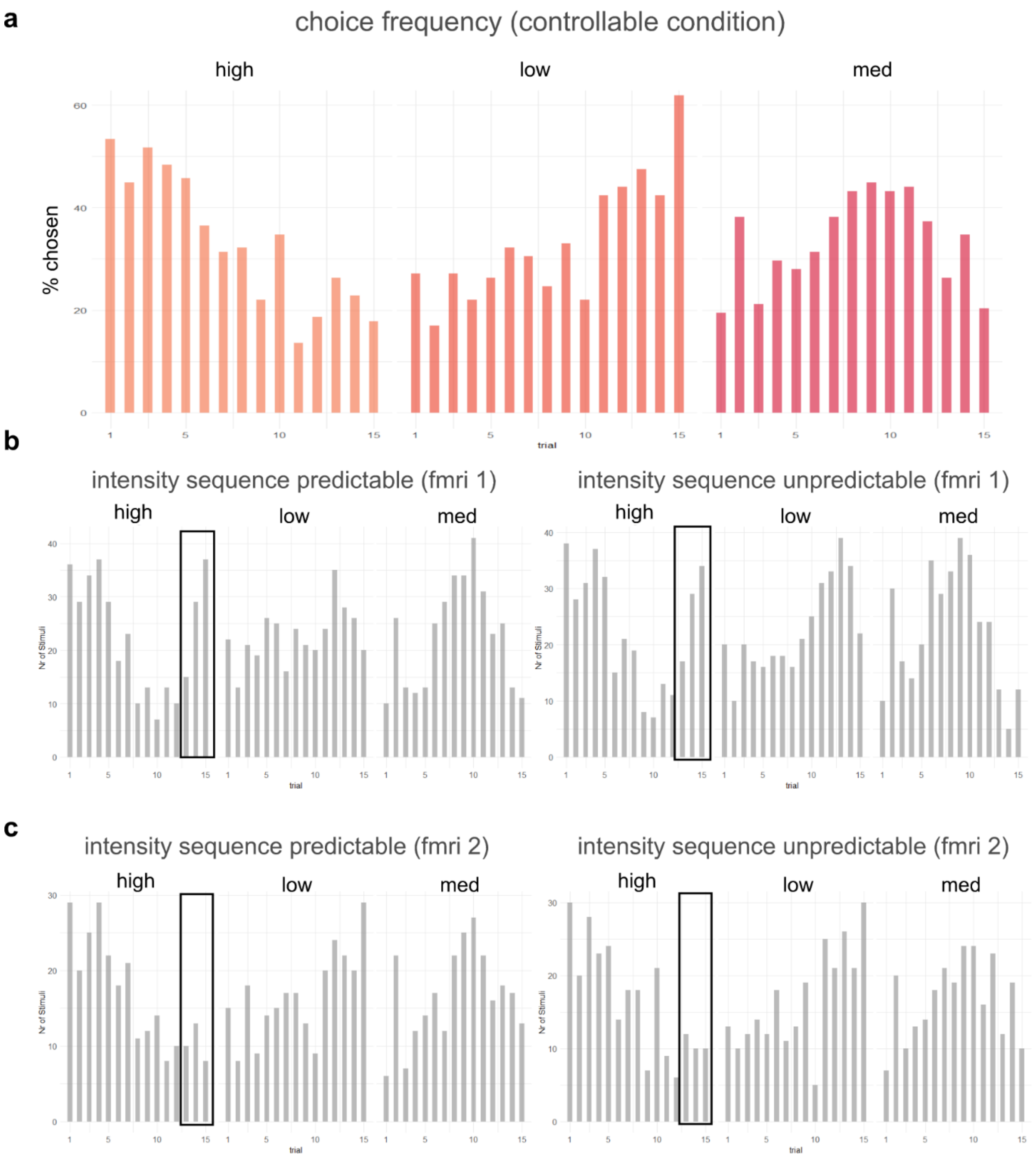

Figure S2 | Choice behavior and intensity sequences in the fMRI sample. (a) Participants showed negative discounting behavior during the controllable condition choosing high painful stimuli at the beginning and keeping low painful stimuli until the end of a run. Participants possibly explored all three intensity levels before settling for a strategy, leading to the relative increase for low and medium intensity in the first three trials **(b)** Informed by the behavioral sample, intensity sequences were adapted for the sample undergoing fMRI scanning. Due to the probabilistic nature of the function, we observed too many high stimuli towards the end of a run in the predictable and unpredictable condition. We adapted the sequence again for the second half of the sample. **(c)** For the second half of the fMRI sample, stimulus intensity sequences were better matched between the controllable, the predictable and the unpredictable condition. There was no increase in high stimuli towards the runs end (see trials marked with the black square in both subsamples).

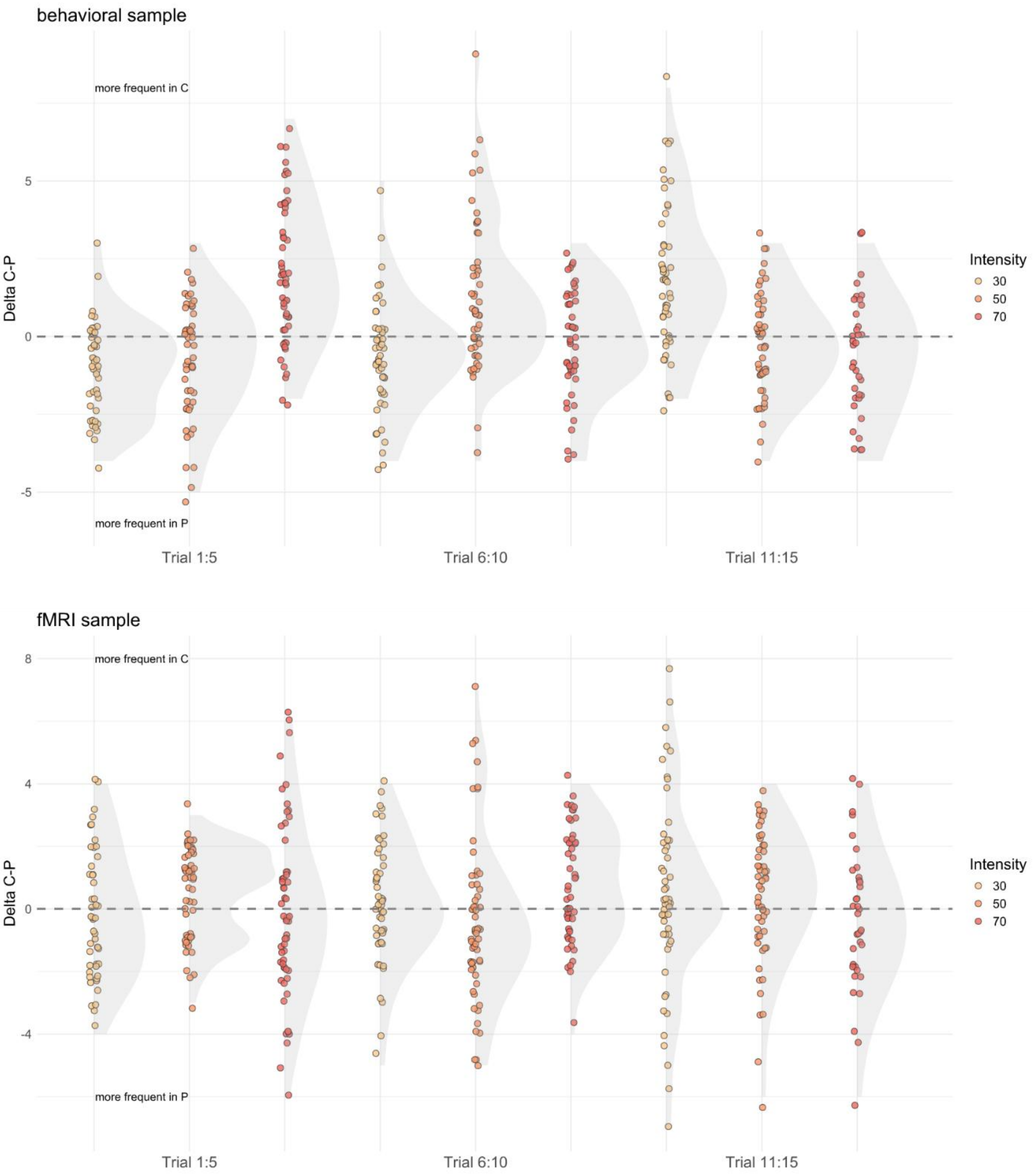

Figure S3| The figure shows the within-participants differences between stimulus intensity frequencies in both runs grouped in three bins of 5 trials, separate for each intensity level. We grouped the data in three time bins (trials 1:5, 6:10, 11:15) and then subtracted, for each time bin the frequency of predictable from controllable stimuli of each intensity level. A positive value indicates a higher occurrence frequency of that intensity level in controllable trials, and a negative value indicates that the intensity level occurred more frequently in predictable trials. As expected, the high intensity was more frequent in the controllable condition in the first time bin, and that the low intensity was more frequent in last third in the behavioral sample. In the fMRI sample, where the frequencies were better matched, we see that in all trial bins the differences in frequency are well distributed across participants and unlikely to have introduced a systematic confounding effect and indicating improvement in matching procedure

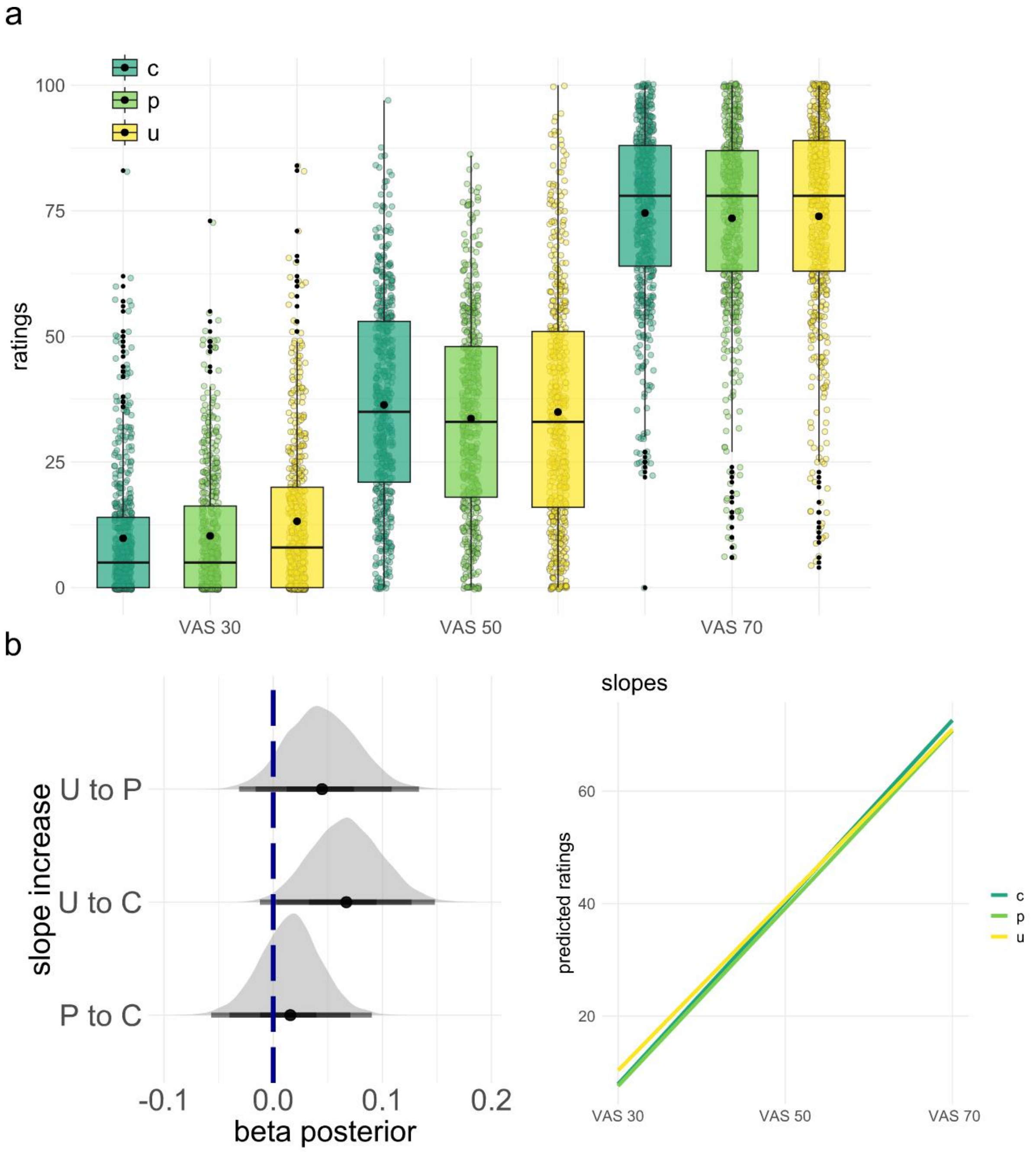

Figure S4| Pain ratings and visualization of interaction effects.

(a) Pain ratings of the behavioral sample (n = 54) in the controllable (C, dark green), predictable (P, light green) and unpredictable (U, yellow) condition. Large black points in the boxplots indicate mean values, boxes show the 25th and 75th percentile around the median line, whiskers extend to ± 1.5* IQR, outliers are shown as small black points. **(b)** Posterior distribution of interaction parameters and fitted lines from the linear model. The grey curves (posteriors) show the estimated probability densities of the parameter value. A positive value of the interaction parameter (beta) indicates a slope increase over intensities for the indicated condition pair, i.e. when moving from the unpredictable to the predictable (U to P), from the unpredictable to the controllable condition (U to C) and from the predictable to the controllable condition (P to C), ratings show a stronger increase from the low to the high intensity level. Posteriors of the interaction parameters are above zero, implying an effect between conditions U and P and U and C. The schematic plot on the right shows the simplified fitted lines derived from model fitting, which illustrate the changes in slope between the controllable, predictable and unpredictable condition.

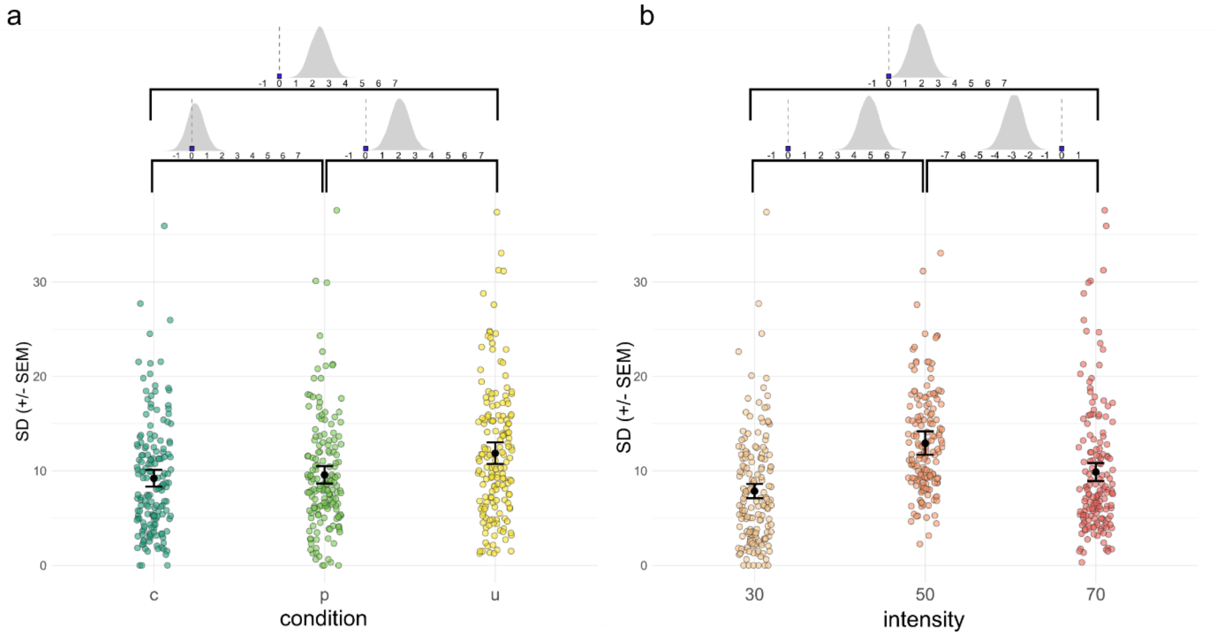

Figure S5 | Within-subject standard deviations of pain ratings for conditions and intensity levels in behavioral sample. (a) Standard deviations of ratings in the controllable condition (dark green) were lower than in the predictable (light green) and the unpredictable (yellow) condition. Black points demarcate the mean standard deviations; error bars the standard error of the mean (SEM) corrected for within-subject measurements. Posteriors of regression parameters (betas) of the pairwise comparisons are displayed above the plots. The grey curves show the estimated probability densities of beta values. If the probability curve is centered at a positive value, this indicates an increase in standard deviation for the condition relative to the reference condition. The value of zero is highlighted on the scale. The posteriors of betas lie above zero for the comparison of the controllable and unpredictable, predictable and unpredictable condition, indicating a substantial influence. **(b)** Standard deviations of pain ratings were highest at the medium intensity level (VAS 50) and higher for the high intensity level (VAS 70) than low intensity level (VAS 30). The posteriors above the plots show the effect magnitude and distribution; the larges fraction and peak of the probability curve are above zero when moving from the low to the medium or the low to the high intensity level. The curve covers negative values when moving from the medium intensity (as baseline) to the high intensity level.

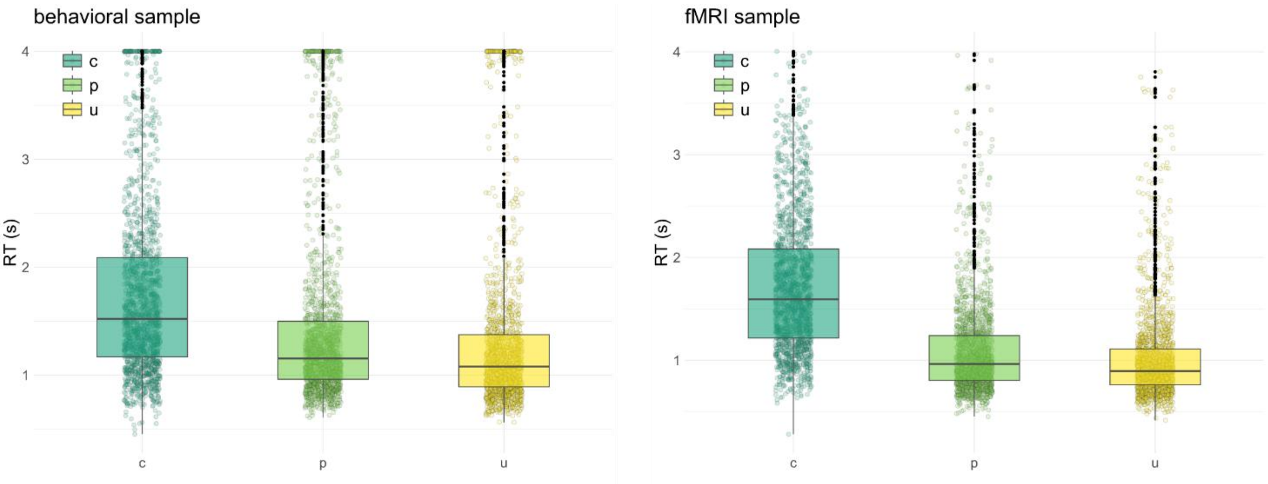

Figure S6 | Reaction times in controllable, predictable and unpredictable trials. In controllable trials, reaction time (RT) refers to the time period before the choice on intensity level was made. In the predictable and unpredictable trials, the RT refers to the time the participants needed to select the correct color from the buttons displayed on the lower part of the screen. RT was significantly higher in controllable than predictable trials and in predictable than unpredictable trials in both samples.

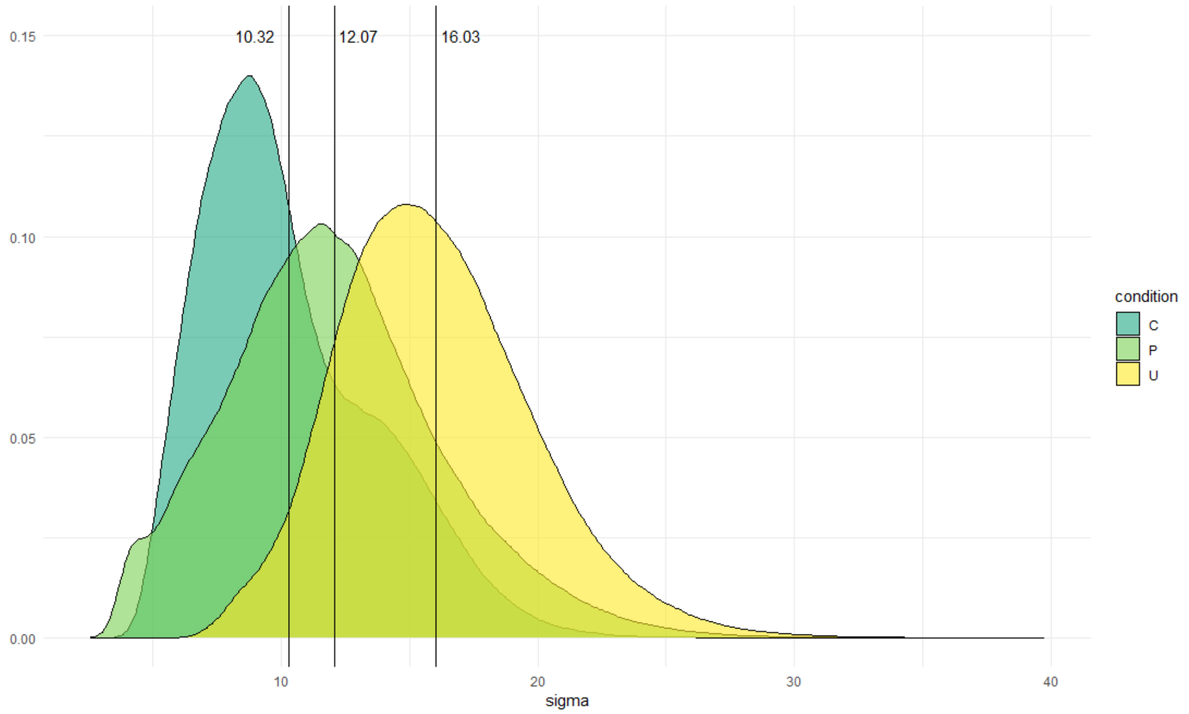

Figure S7|Posterior of prior uncertainty parameter. The standard deviation is lowest in the controllable condition, followed by the predictable and the unpredictable condition. It can be interpreted as a proxy of prior uncertainty, as the sensory input was kept equal in all conditions.

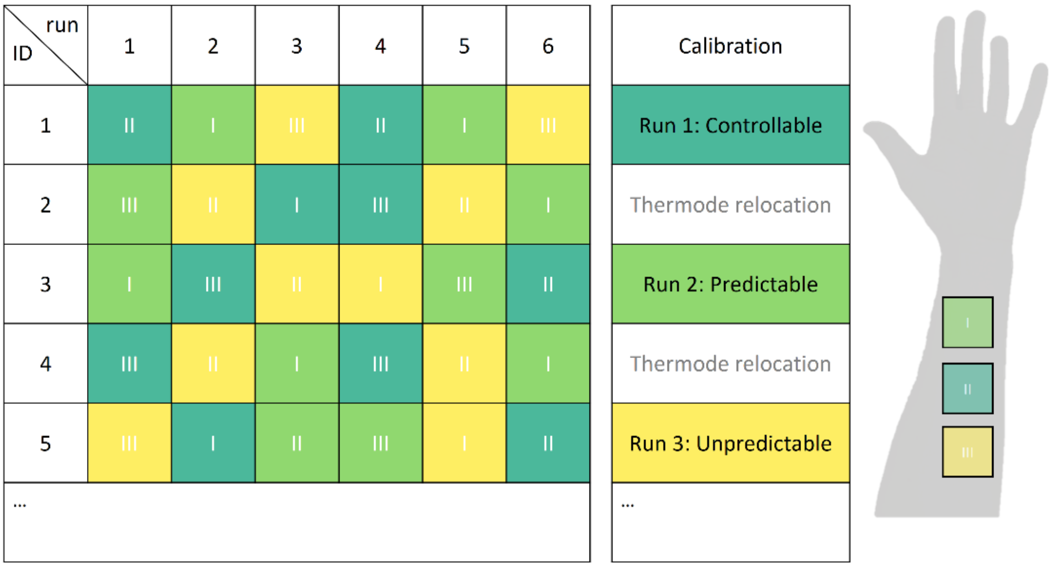

Figure S8| Exemplary condition (dark green: controllable, light green: predictable, yellow: unpredictable) and thermode location (roman numbers I-III) randomization schedule for 5 participants. Conditions were nested into runs (1-6). After calibration, six runs followed in two randomized triplets separated by a short break for thermode replacement. Each location was stimulated twice throughout the experiment. Thermode location sequence on the arm was randomized independently of condition. The same sequence as in runs 1-3 was repeated in run 4-6 to keep breaks between skin patches equally long. Each run took approximately 7 minutes. After the calibration the location of the thermode was not changed. Before each run a VAS 50 stimulus was applied for pre-exposure. In runs 1-3 participants did 3 test trials of the new condition. If they had difficulties or questions they had the possibility to repeat the test trials. No test trials were added for runs 4-6, because by then the participants were already familiar with the different tasks.

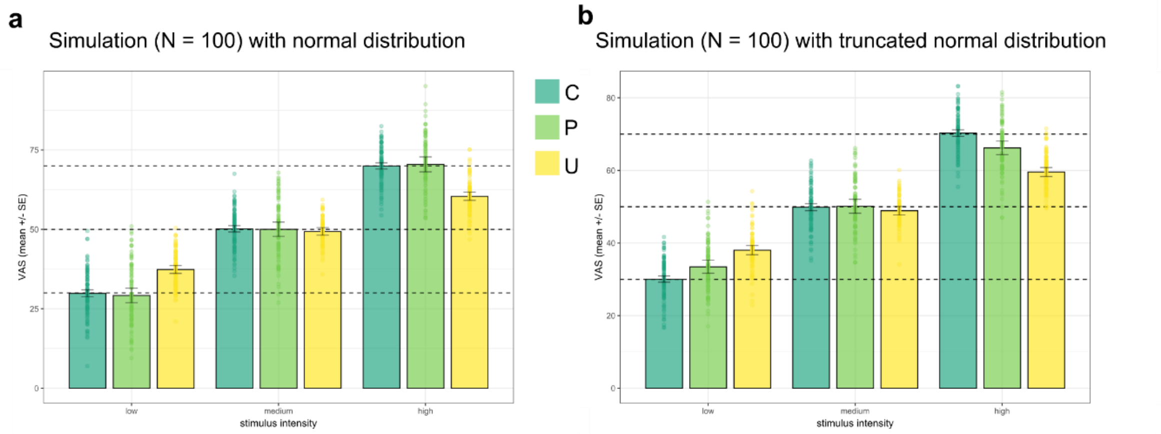

Figure S9 |Simulated data with normal distributions (left) and truncated normal distributions (right) for the sampling process. When comparing the same normal-normal integration process in the Bayesian perception framework a change in prior precision would normally not lead to differences in posterior means, if the means of the priors and the likelihood distribution remain the same. But because pain ratings have a lower and an upper boundary (pain threshold and pain tolerance) they can be appropriately modelled with truncated normal distributions, ranging from 0 to 100. With this hard cut, different distribution precisions lead to the interactive data pattern. For the simulations, the standard deviations were set to $SD_{controllable}$ = 5 and $SD_{predictable}$ = 20, for visualization purpose. 100 datasets were simulated, all with 90 runs for each simulated subject. The prior mean in the unpredictable condition was the weighted mean of all possible outcome values. Prior means were set to the VAS targets 30, 50 and 70 in all conditions and equal in both simulations. We simulated one dataset with a normal distribution without boundaries and once with a truncated normal distribution. **(a)** Shows the outcome with normal distribution without boundaries. C and P have the same mean value despite the different prior precisions. **(b)** The right side matches the true data pattern, i.e. the interaction effect between C and P across intensities.

model with 3 scaling parameter "alpha" (dynamic)

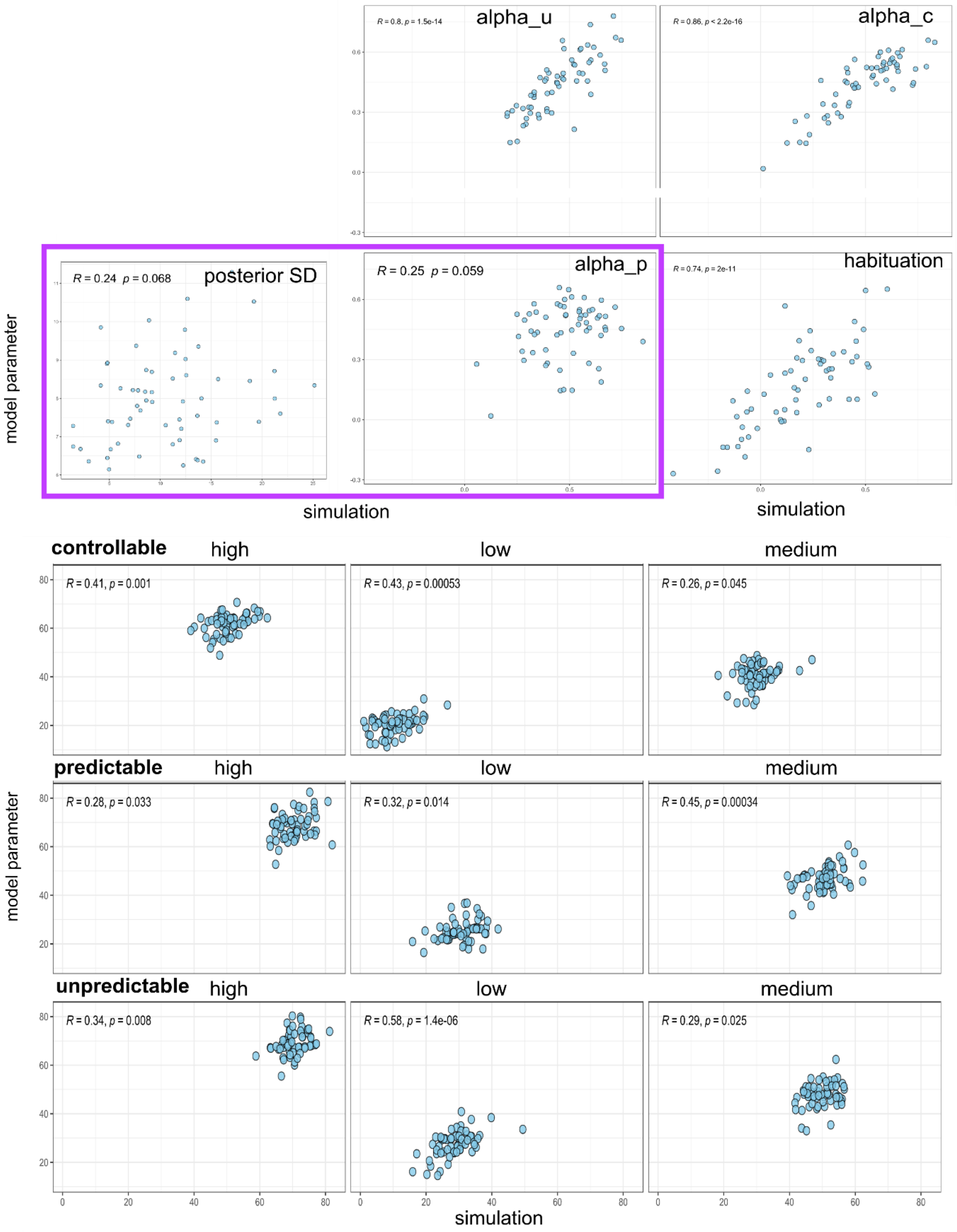

Figure S10 | Parameter recovery results of the model with three alpha scaling parameters. The parameters highlighted in violet could not be recovered sufficiently well in relation to their importance for the model. Therefore, we could not proceed with the model with three scaling parameters (alpha_U, alpha_C, alpha_P), see Method section.

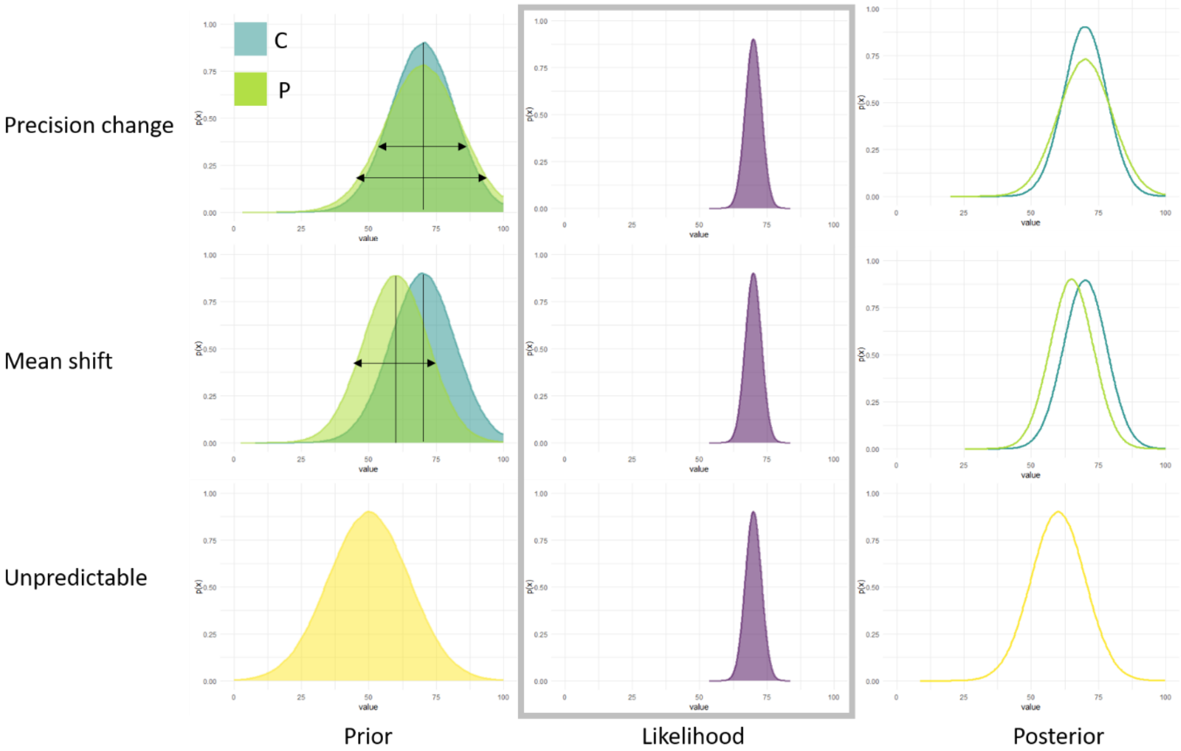

Figure S11 | Schematic display of the model distributions in for high intensity trials and the precision-change and the mean-shift model. In the precision change model, one value is sampled for the prior means of the controllable and predictable condition, while the precision can be different. Note that because the posterior in the predictable condition has thicker tails and is truncated at VAS 100, a decrease of ratings in the predictable condition for high intensity stimuli can result from this model. In the mean shift model, only one value for the precision of the prior can be sampled for each participant to account for the controllable and predictable condition, but the prior means are flexible. The prior in the unpredictable condition is wider than in the other conditions and centered on the middle of the VAS scale. The likelihood is assumed to be constant in all conditions.

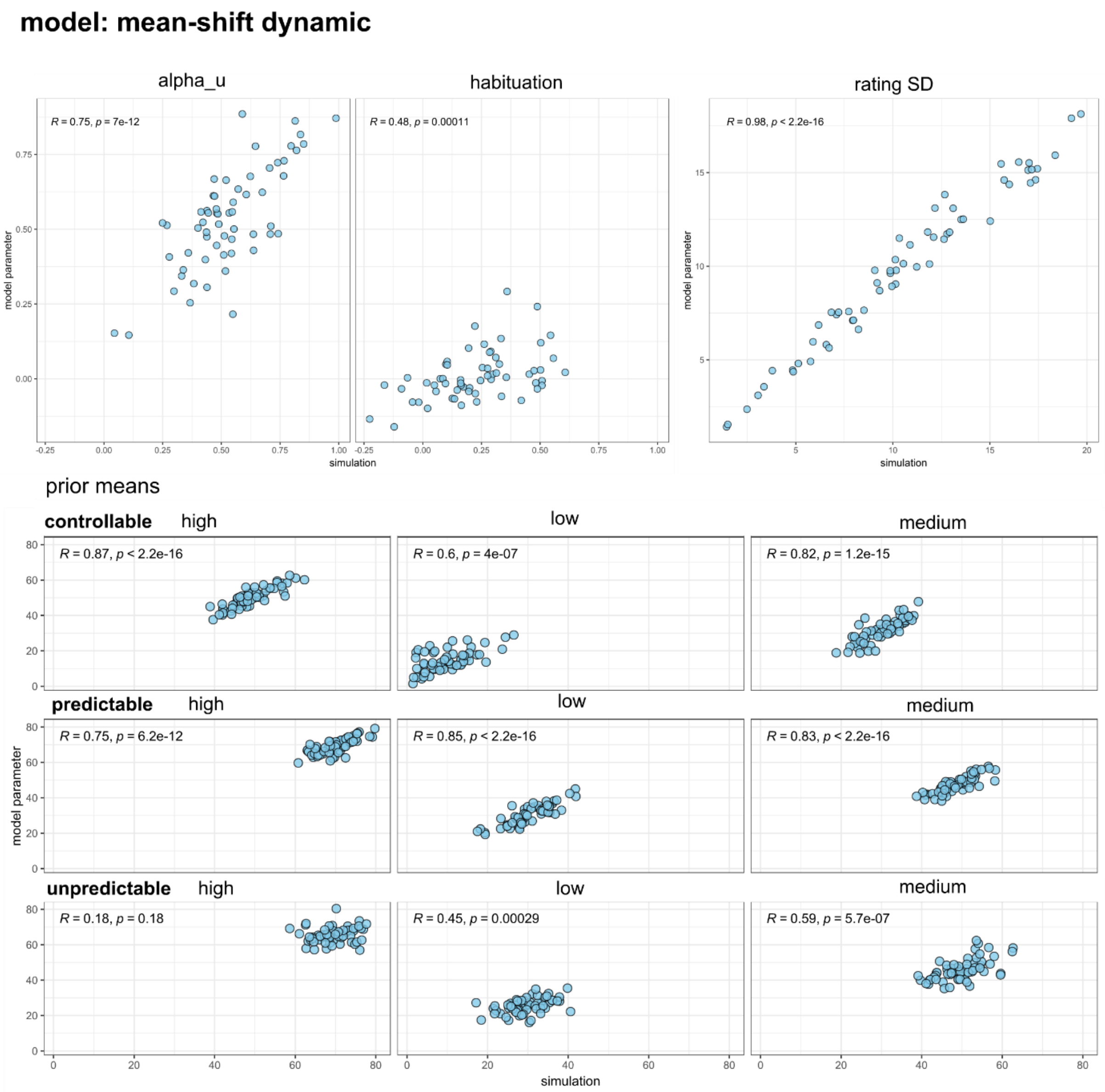

Figure S12| Parameter recovery results of the mean-shift model. All parameter can be reasonably well recovered (significant correlation, visual inspection). It was more difficult to recover the prior means of the unpredictable condition due to the weighting of means implemented in the model. The standard deviations of the priors in the controllable and predictable condition were recovered very well.

model: precision-change dynamic

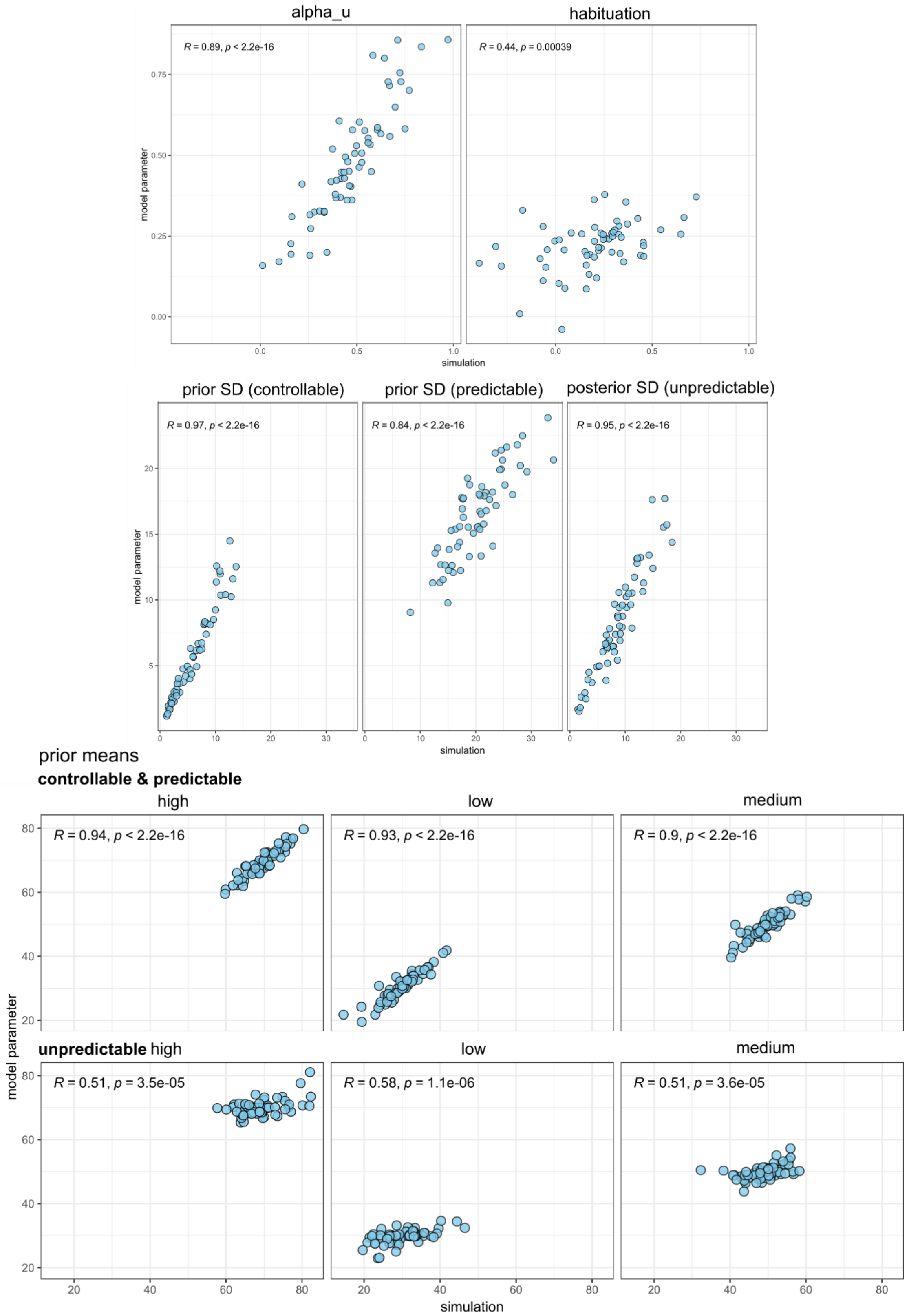

Figure S13 | Parameter recovery results of the winning precision-change model. All parameter can be reasonably well recovered (significant correlation, visual inspection). It was more difficult to recover the prior means of the unpredictable condition due to the weighting of means implemented in the model. The standard deviations of the priors in the controllable and predictable condition were recovered very well.

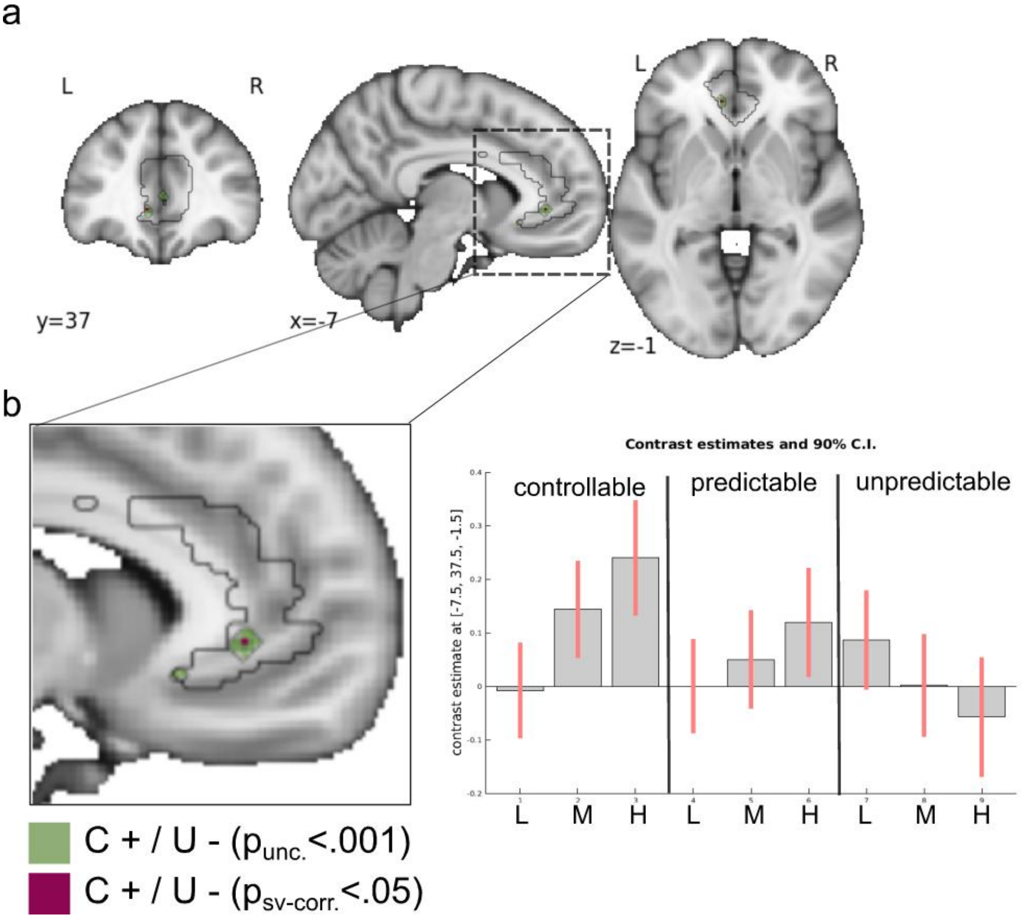

Figure S14 | Interaction effects in ACC (a) Thresholded statistical maps for the interaction effect between controllable and unpredictable condition, masked with an ACC-mask, which was also used for (post-hoc) small volume correction. Green areas indicate regions significant at uncorrected p <.001. The pink region show the cluster that would be significant with small volume correction using the outlined ACC mask. **(b)** Parameter estimates of the significant interaction voxel, visualized with a 9-regressors model separately showing activity in the voxel for the three different task conditions at low (L), medium (M) and high (H) stimulus intensity. While there is a positive scaling with intensity for controllable and predictable condition, activity decreases with intensity in the unpredictable condition.

a

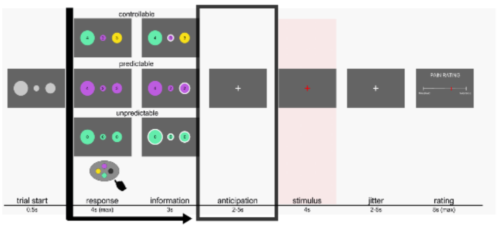

b

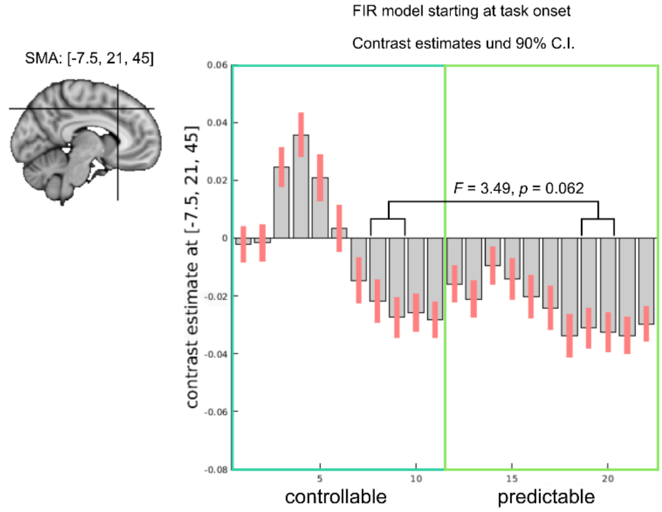

c

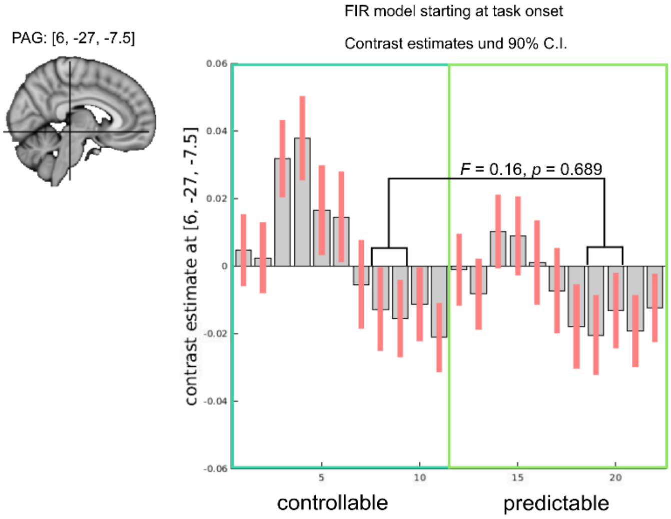

Figure S15 | Parameter estimates derived from an FIR model of the decision task. To investigate possible influence of the different tasks (intensity choice vs. color-matching task) in terms of cognitive demand, we modeled the task with finite-impulse response (FIR) models, starting at task onset and covering the time until stimulus onset. We evaluated differences between conditions at task and at the time bins relevant for pain onset by contrasting activity at the relevant time bins (i.e. bins 8 & 9 covering 10.5-13.5s). Please note that the temporal gap between the choice period and the trigger impulse to start the thermode was in total 9-12s (4s choice, 3s information and 2-5s anticipation) plus an additional delay of ~1s until the thermode reached the desired temperature (0.89s for VAS 30, 1.02s for VAS 50 and 1.16s for VAS 70) leading to an effective stimulus onset of 10-13s. **(a)** Shows the time period covered by the FIR model in the schematic task figure: 11 TR of 1.5s, resulting in 16.5s in total. Time courses are shown for the peak voxels in **(b)** SMA and **(c)** PAG, where we find a significant reduction in the controllable condition compared to the predictable condition during pain (see Figure 5b).

Supplemental Table 1: Choice behavior

Results of linear models on choice frequency (model parameters and convergence measures)

Model	Param.	*M*	*SE*	*SD*	*25%*	*50%*	*97%*	$N_{eff.}$	$\widehat{R}$	$HPDI_{min}$	$HPDI_{max}$	Int.	Sample
Lin.	α	8.66	0.07	4.13	6.06	8.58	17.07	3825.89	1	0.36	16.82	L	1
	β	3.08	0.01	0.45	2.80	3.09	3.96	3785.79	1	2.18	3.96	L	1
	ε	7.22	0.02	1.62	6.08	6.95	11.15	4513.15	1	4.52	10.38	L	1
Lin.	α	15.64	0.07	4.54	12.79	15.67	24.71	3798.80	1	6.59	24.73	L	2
	β	2.21	0.01	0.50	1.91	2.21	3.20	3914.47	1	1.19	3.19	L	2
	ε	8.05	0.03	1.85	6.75	7.74	12.62	4155.37	1	4.98	11.74	L	2
Lin.	α	29.52	0.10	6.50	25.34	29.48	42.92	3906.07	1	17.38	43.34	M	1
	β	0.48	0.01	0.71	0.05	0.50	1.85	4138.02	1	-0.92	1.92	M	1
	ε	11.84	0.04	2.69	9.98	11.36	18.38	3839.43	1	7.56	17.31	M	1
Lin.	α	29.95	0.08	5.58	26.43	29.94	41.26	4410.20	1	18.81	41.05	M	2
	β	0.43	0.01	0.61	0.05	0.43	1.60	4388.21	1	-0.79	1.61	M	2
	ε	10.05	0.04	2.29	8.44	9.68	15.54	4172.20	1	6.10	14.40	M	2
Poly.	α	10.27	0.16	8.16	5.19	10.36	26.52	2711.77	1	-6.41	26.34	M	1
	β1	7.26	0.05	2.35	5.80	7.25	12.11	2528.42	1	7.28	7.28	M	1
	β2	-0.42	0.00	0.14	-0.51	-0.42	-0.14	2675.59	1	9.38	9.38	M	1
	ε	8.73	0.03	2.06	7.30	8.36	13.81	3616.42	1	5.44	12.93	M	1
Poly.	α	13.62	0.13	6.77	9.33	13.58	27.29	2867.81	1	0.28	27.08	M	2
	β1	6.18	0.04	1.95	4.97	6.19	10.02	2662.75	1	9.67	9.67	M	2
	β2	-0.36	0.00	0.12	-0.43	-0.36	-0.12	2838.70	1	5.98	5.98	M	2
	ε	7.43	0.03	1.77	6.20	7.13	11.73	3392.04	1	4.49	10.92	M	2
Lin.	α	62.07	0.06	4.14	59.47	62.05	70.37	4512.37	1	53.43	69.97	H	1
	β	-3.59	0.01	0.46	-3.88	-3.60	-2.68	4439.29	1	-4.45	-2.65	H	1
	ε	7.53	0.02	1.65	6.36	7.26	11.51	4661.54	1	4.67	10.74	H	1
Lin.	α	54.33	0.06	3.78	51.97	54.34	61.80	3982.86	1	46.87	61.94	H	2
	β	-2.63	0.01	0.41	-2.89	-2.63	-1.79	3935.79	1	-3.42	-1.76	H	2
	ε	6.70	0.02	1.49	5.63	6.47	10.28	4782.92	1	4.21	9.66	H	2

Note. Int. = intensity; L = low intensity, M = medium intensity, H = high intensity; Sample 1 = behavioral sample, Sample 2 = fMRI sample. α = intercept, β = slope, ε = error. The sign of β indicates either an increase or decrease of frequency of the intensity level over trials (controllable condition only). Models were run on percentage values of each intensity level per trial on group level.

Supplemental Table 2: Pain ratings

Results of linear mixed models on pain ratings (model parameters and convergence measures)

Sample	Param.	M	SE	SD	25%	50%	97%	$N_{eff.}$	$\hat{R}$	$HPDI_{min}$	$HPDI_{max}$
1	α	-33.13	0.08	1.91	-34.41	-33.11	-29.34	589.01	1.01	-37.00	-29.42
1	trial	-0.19	0.00	0.05	-0.22	-0.19	-0.09	8802.15	1.00	-0.29	-0.09
1	sess.	-1.38	0.00	0.13	-1.47	-1.38	-1.13	8499.79	1.00	-1.64	-1.13
1	int.	1.59	0.00	0.02	1.58	1.59	1.64	2804.69	1.00	1.55	1.64
1	IA CP	-0.02	0.00	0.03	-0.03	-0.02	0.04	2781.57	1.00	-0.07	0.04
1	IA CU	-0.07	0.00	0.03	-0.09	-0.07	-0.01	3028.21	1.00	-0.13	0.00
1	IA PU	-0.04	0.00	0.03	-0.07	-0.04	0.02	2746.76	1.00	-0.11	0.02
1	ε (sub)	10.39	0.01	1.07	9.62	10.30	12.72	8036.51	1.00	8.45	12.53
1	ε	15.46	0.00	0.16	15.36	15.46	15.77	10478.41	1.00	15.15	15.76
2	α	-30.32	0.05	1.73	-31.50	-30.30	-26.90	1244.23	1	-33.72	-26.94
2	trial	-0.40	0.00	0.05	-0.43	-0.40	-0.31	10503.77	1	-0.50	-0.30
2	sess.	-0.36	0.00	0.12	-0.45	-0.36	-0.13	9309.97	1	-0.60	-0.13
2	int.	1.45	0.00	0.02	1.43	1.45	1.49	3046.05	1	1.40	1.49
2	IA CP	-0.08	0.00	0.03	-0.10	-0.08	-0.02	3476.59	1	-0.14	-0.02
2	IA CU	-0.29	0.00	0.03	-0.31	-0.29	-0.23	3368.49	1	-0.35	-0.23
2	IA PU	-0.20	0.00	0.03	-0.22	-0.20	-0.14	3080.84	1	-0.27	-0.14
2	ε (sub)	7.95	0.01	0.79	7.40	7.89	9.66	9553.99	1	6.49	9.54
2	ε	15.18	0.00	0.15	15.08	15.18	15.48	12770.01	1	14.88	15.47

Note. Main and interaction (IA) effects of the linear model analysis of pain ratings. Fixed effect predictors: trial; sess.: session; Int.: intensity, U: unpredictable, C: controllable; P: predictable; Main effects of condition are omitted, as they are not meaningful in this model with significant interaction effects. Random effect: ε (sub). ε: noise. IA indicates the pairwise interaction of the conditions over intensity levels. The first condition indicates the reference condition for the interaction effect. For visual depiction, direction of the interaction effects is reported in the opposite direction (not the slope decrease from C to P and U, but slope increase from U to C and P, and C to P), the effect is equivalent. HPDIs were computed for 95% of the probability mass of the posterior. Sample 1 = behavioral sample, Sample 2 = fMRI sample. α = intercept

Supplemental Table 3: Standard deviations of pain ratings

Results of linear mixed models on standard deviations pain ratings (model parameters and convergence measures)

Sample	Param.	*M*	*SE*	*SD*	*25%*	*50%*	*97%*	$N_{eff.}$	$\hat{R}$	$HPDI_{min}$	$HPDI_{max}$
1	α	9.47	0.01	0.68	9.02	9.47	10.79	6963.74	1	8.19	10.83
1	β (UC)	-2.45	0.00	0.59	-2.84	-2.44	-1.31	15607.34	1	-3.57	-1.30
1	β (UP)	-2.09	0.00	0.58	-2.48	-2.08	-0.95	16189.86	1	-3.29	-1.00
1	β (LM)	4.91	0.00	0.60	4.50	4.91	6.07	15340.13	1	3.76	6.09
1	β (LH)	1.84	0.00	0.60	1.44	1.84	3.01	15894.08	1	0.68	3.01
1	ε (sub)	2.94	0.00	0.40	2.66	2.92	3.78	9652.42	1	2.19	3.74
1	ε	5.32	0.00	0.18	5.19	5.31	5.69	20729.85	1	4.97	5.68
1	β (CP)	0.27	0.00	0.57	-0.10	0.26	1.40	17474.73	1	-0.87	1.35
1	β (MH)	-2.87	0.00	0.59	-3.26	-2.87	-1.70	16010.91	1	-4.09	-1.76
2	α	12.06	0.01	0.57	11.67	12.05	13.19	9968.29	1	10.93	13.17
2	β (UC)	-4.41	0.00	0.53	-4.77	-4.40	-3.37	18069.93	1	-5.44	-3.36
2	β (UP)	-3.31	0.00	0.53	-3.67	-3.30	-2.29	17610.12	1	-4.33	-2.29
2	β (LM)	3.98	0.00	0.53	3.61	3.98	5.00	17626.36	1	2.96	5.02
2	β (LH)	2.32	0.00	0.52	1.97	2.32	3.37	17334.42	1	1.32	3.38
2	ε (sub)	2.34	0.00	0.34	2.10	2.32	3.05	7324.10	1	1.69	3.01
2	ε	4.94	0.00	0.16	4.83	4.94	5.27	20180.26	1	4.63	5.27
2	β (CP)	0.98	0.00	0.51	0.65	0.98	1.99	18011.92	1	0.02	2.04
2	β (MH)	-1.50	0.00	0.52	-1.85	-1.50	-0.48	17098.70	1	-2.49	-0.46

Note. Pairwise effects of conditions and intensity levels, resulting from the linear model analysis on standard deviation of pain ratings. Fixed effect predictors: U: unpredictable, C: controllable; P: predictable; L: low intensity; M: medium intensity; H: high intensity. Random effect: ε (sub). ε: noise. The first condition in braces indicates the reference condition/intensity. HPDIs were computed for 95% of the probability mass of the posterior. Sample 1 = behavioral sample, Sample 2 = fMRI sample. α = intercept

Supplemental Table 4: Robustness analysis of pain ratings with LME4

Summary of results from LMMs on pain ratings as implemented in LME4

	β	SE	df	t	p	sample	N
α	-32.32	1.32	612.20	-24.55	0.00	combined	113
trial	-0.26	0.04	10050.00	-7.25	0.00	combined	113
session	-0.85	0.09	10050.00	-9.47	0.00	combined	113
IA CP	-0.06	0.02	10050.00	-2.61	0.01	combined	113
IA CU	-0.19	0.02	10050.00	-8.44	0.00	combined	113
α	-33.39	2.02	229.53	-16.51	0.00	behavioral	54
trial	-0.18	0.05	4799.00	-3.50	0.00	behavioral	54
session	-1.39	0.13	4799.00	-10.68	0.00	behavioral	54
IA CP	-0.02	0.03	4799.00	-0.62	0.53	behavioral	54
IA CU	-0.08	0.03	4799.00	-2.49	0.01	behavioral	54
α	-30.62	1.69	406.01	-18.08	0.00	fMRI	59
trial	-0.40	0.05	5244.00	-8.08	0.00	fMRI	59
session	-0.37	0.12	5244.00	-3.03	0.00	fMRI	59
IA CP	-0.10	0.03	5244.00	-3.20	0.00	fMRI	59
IA CU	-0.30	0.03	5244.00	-9.67	0.00	fMRI	59
α	-27.62	2.20	217.53	-12.56	0.00	fMRI (1)	34
trial	-0.43	0.06	3019.00	-6.76	0.00	fMRI (1)	34
session	-0.29	0.16	3019.00	-1.86	0.06	fMRI (1)	34
IA CP	-0.06	0.04	3019.00	-1.48	0.14	fMRI (1)	34
IA CU	-0.25	0.04	3019.00	-6.34	0.00	fMRI (1)	34
α	-35.03	2.67	172.89	-13.14	0.00	fMRI (2)	25
trial	-0.35	0.08	2218.00	-4.45	0.00	fMRI (2)	25
session	-0.43	0.19	2218.00	-2.21	0.03	fMRI (2)	25
IA CP	-0.15	0.05	2218.00	-3.13	0.00	fMRI (2)	25
IA CU	-0.37	0.05	2218.00	-7.42	0.00	fMRI (2)	25

Note. Main effects for condition, intensity are omitted as they are only based on the lowest level of the predictors that are included in the interaction term and therefore not generalizable to the other levels of the interacting factor. α = intercept. Fixed effect predictors: trial; session; U: unpredictable, C: controllable; P: predictable; Random effect: ε (sub). ε: noise. IA indicates the pairwise interaction of the conditions over intensity levels. The first condition indicates the reference condition for the interaction effect. P-values for LMMs were estimated using Satterthwaite's method for approximating degrees of freedom with lmerTest package, tests are two-sided.

Supplemental Table 5: Interaction analysis of two subsamples of fMRI sample

Results of interaction analysis

	β	SE	df	t	p	N
α	-27.78	2.15	338.14	-12.90	0.00	59
trial	-0.40	0.05	5239.00	-8.01	0.00	59
session	-0.35	0.12	5239.00	-2.86	0.00	59
conditionp:intensity:groupfmri_2	-0.09	0.06	5239.00	-1.48	0.14	59
conditionu:intensity:groupfmri_2	-0.11	0.06	5239.00	-1.75	0.08	59

Note. Main effects for condition, intensity and group are omitted as they are only based on the lowest level of the predictors that are included in the interaction term and therefore not generalizable to the other levels of the interacting factor. α = intercept. Reference conditions are: fmri group 1, controllable condition, intensity 0. P-values for LMMs were estimated using Satterthwaite's method for approximating degrees of freedom with lmerTest package, tests are two-sided.

Supplemental Table 6: Stimulus intensity levels and average ratings

Pain ratings and Temperatures for all (Sub-)Samples

Sample	VAS target	Rating		Temperature	
		M	*SD*	*M*	*SD*
behavioral	0	-	-	40.93	4.25
behavioral	30	11.12	13.73	43.56	2.63
behavioral	50	35.00	21.15	45.31	1.66
behavioral	70	74.01	19.14	47.07	1.14
behavioral	100	-	-	49.76	2.05
fmri (1)	0	-	-	41.70	3.15
fmri (1)	30	14.30	14.84	43.97	2.20
fmri (1)	50	36.57	18.66	45.49	1.69
fmri (1)	70	66.51	16.77	46.98	1.32
fmri (1)	100	-	-	49.28	1.48
fmri (2)	0	-	-	42.62	2.37
fmri (2)	30	12.12	14.27	44.77	1.70
fmri (2)	50	34.89	19.90	46.21	1.52
fmri (2)	70	68.11	17.95	47.50	1.40
fmri(2)	100	-	-	49.81	2.17

Note. fmri (1) and (2) refer to the samples with different intensity sequence generation. Stimuli at threshold (VAS 0) or tolerance level (VAS 100) were not applied throughout the experiment therefore no rating data is available for these intensity levels.

Supplemental Table 7: Demographic information

Demographic Information and Questionnaire Results

Sample	N	Age		STAI		LC internal		LC external	
		M	*SD*	*M*	*SD*	*M*	*SD*	*M*	*SD*
Behavioral	54	27.15	4.54	36.59	7.06	56.76	16.86	51.39	16.54
fMRI	59	26.56	5.11	33.14	6.36	67.15	9.01	46.93	10.00

Note. STAI: State-Trait Anxiety Inventory (State Form); LC internal: Score of internal locus of control assessed with *Fragebogen zu Kompetenz- und Kontrollüberzeugungen* (FKK), a German version *of Internal/External Locus of Control Scale*; LC external: Score of external locus of control assessed with FKK.

Parameter recovery

Computational models applied to behavioral data are supposed to reveal hidden parameters that are influencing overt behavior. To be sure that a model can actually perform as we want it to, one needs to be sure that parameters are correctly recovered by the model. We simulated data employing the null models and models of interest that were later applied to the experimental data of two studies. We provide the results of the parameter recovery for the nullmodels, the model with three different scaling parameters, the mean-shift and the precision change model with a dynamic prior mean in the unpredictable condition. For the interested reader, the parameter recovery and simulation scripts for all other model versions can run with the code provided in the associated repository.

Data was simulated with N = 60 participants with 6 sessions, resulting in 90 trials in total for each simulated participant and model type (see all input values for the simulation in Supplemental Table 8). We visually inspected the simulated data patterns as a sanity check. Then the different model versions were fitted to the simulated data and parameter values that resulted from the fit were correlated against parameter values that entered the simulation. As a measure of validity we examined scatterplots, report *p*-values and *R*-values of the correlation (see Figures S8, S10, S11). The points in the scatterplots show the mean value of the simulated subject posterior of the model parameter.

Supplemental Table 8
Simulation settings

	**Input values**
**number of subjects**	60
**number of trials**	15
**number of sessions**	6
**likelihood mean**	$\text{mean} \in \{30, 50, 70\}, \text{sd} = 1$
**prior mean controllable**	$\text{mean} \in \{10, 30, 50\}, \text{sd} = 15$
**prior mean predictable**	$\text{mean} \in \{30, 50, 70\}, \text{sd} = 15$
**prior mean unpredictable**	$\text{mean} \in \{30, 50, 70\}, \text{sd} = 15$
**h**	$\text{mean} = 0.5, \text{sd} = 0.2$
**Posterior SD**	$\text{mean} = 10, \text{sd} = 5$
**prior SD C**	$\text{mean} = 10, \text{sd} = 5$
**prior SD P**	$\text{mean} = 10, \text{sd} = 5$
**alpha_U**	$\text{mean} = 0.5, \text{sd} = 0.2$
**alpha_C**	$\text{mean} = 0.5, \text{sd} = 0.2$
**alpha_P**	$\text{mean} = 0.5, \text{sd} = 0.2$

Note. All simulation input value parameters were sampled from truncated normal distributions around the specified mean and standard deviation. For likelihood and prior means the distributions had a lower bound at 0 and an upper bound at 100. The other parameters were constrained to lie in the interval [0,1].
