# Supplementary material for: Controllability changes pain perception by increasing the precision of expectations
Source: Nat Commun. 2025 Nov 18;16:10113. doi: 10.1038/s41467-025-66038-7 (PMC12627469; doi:10.1038/s41467-025-66038-7)
Supplement: Supplementary file 2 — Reporting Summary [file 41467_2025_66038_MOESM2_ESM.pdf]

nature portfolio

Corresponding author(s): Marie Habermann

Last updated by author(s): Sep 22, 2025

Reporting Summary

Nature Portfolio wishes to improve the reproducibility of the work that we publish. This form provides structure for consistency and transparency in reporting. For further information on Nature Portfolio policies, see our Editorial Policies and the Editorial Policy Checklist.

Statistics

For all statistical analyses, confirm that the following items are present in the figure legend, table legend, main text, or Methods section.

n/a	Confirmed	
☐	☒	The exact sample size (*n*) for each experimental group/condition, given as a discrete number and unit of measurement
☐	☒	A statement on whether measurements were taken from distinct samples or whether the same sample was measured repeatedly
☐	☒	The statistical test(s) used AND whether they are one- or two-sided<br>*Only common tests should be described solely by name; describe more complex techniques in the Methods section.*
☐	☒	A description of all covariates tested
☐	☒	A description of any assumptions or corrections, such as tests of normality and adjustment for multiple comparisons
☐	☒	A full description of the statistical parameters including central tendency (e.g. means) or other basic estimates (e.g. regression coefficient) AND variation (e.g. standard deviation) or associated estimates of uncertainty (e.g. confidence intervals)
☐	☒	For null hypothesis testing, the test statistic (e.g. *F*, *t*, *r*) with confidence intervals, effect sizes, degrees of freedom and *P* value noted<br>*Give P values as exact values whenever suitable.*
☐	☒	For Bayesian analysis, information on the choice of priors and Markov chain Monte Carlo settings
☒	☐	For hierarchical and complex designs, identification of the appropriate level for tests and full reporting of outcomes
☒	☐	Estimates of effect sizes (e.g. Cohen's *d*, Pearson's *r*), indicating how they were calculated

Our web collection on statistics for biologists contains articles on many of the points above.

Software and code

Policy information about availability of computer code

Data collection	Code for the behavioral experiments was written in MATLAB2020a using Psychtoolbox 3.
Data analysis	The behavioral data were preprocessed and analyzed using MATLAB2020a, R. Computational models were run using R and Stan (rstan). The fMRI data were analyzed using MATLAB2020a and SPM12. Analysis code is available at: https://gin.g-node.org/mahabermann/copain_code.git

For manuscripts utilizing custom algorithms or software that are central to the research but not yet described in published literature, software must be made available to editors and reviewers. We strongly encourage code deposition in a community repository (e.g. GitHub). See the Nature Portfolio guidelines for submitting code & software for further information.

Data

Policy information about availability of data

All manuscripts must include a data availability statement. This statement should provide the following information, where applicable:

- Accession codes, unique identifiers, or web links for publicly available datasets
- A description of any restrictions on data availability
- For clinical datasets or third party data, please ensure that the statement adheres to our policy

Behavioral and fMRI data is available at following address: https://gin.g-node.org/mahabermann/copain_data.git

Research involving human participants, their data, or biological material

Policy information about studies with human participants or human data. See also policy information about sex, gender (identity/presentation), and sexual orientation and race, ethnicity and racism.

Reporting on sex and gender	Gender was assessed based on self-reporting with options "male", "female", "diverse", "n/a" on a computerized form. In sample 1, 33 participants self-identified as female, 25 participants as male, and one person preferred not disclose the information. In sample 2, 41 participants self-identified as female and 28 as male. No sex- or gender-based analyses were performed because we did not expect systematic differences between the groups.
Reporting on race, ethnicity, or other socially relevant groupings	This information has not been collected.
Population characteristics	Sample 1: mean age = 26.8, range 19 to 39. Participants were included if they met none of the exclusion criteria: acute or chronic somatic or psychiatric disease, drug or medication intake (except oral contraception, allergy & thyroid medication), chronic or acute pain condition.<br><br>Sample 2: mean age = 26.26, range 18 to 40. In addition to the exclusion criteria above participants were screened for MRI contraindications (e.g. pregnancy, metal implants, claustrophobia).
Recruitment	Participants were recruited via a local online platform (www.stellenwerk.de/hamburg). Screening for exclusion criteria was completed via e-mail and phone. All participants provided informed consent and were paid for participation (10€/h for the behavioral study, 15€/h for the MRI study). Only people who visited the online platform could find information about the study. We see no reason to assume that this procedure had a major effects on the results or introduced effects due to self-selection bias.
Ethics oversight	The local ethics committee (Ethikkommission der Ärztekammer Hamburg) approved the study (ref.nr.: 2020-10230-BO-ff).

Note that full information on the approval of the study protocol must also be provided in the manuscript.

Field-specific reporting

Please select the one below that is the best fit for your research. If you are not sure, read the appropriate sections before making your selection.

☒ Life sciences ☐ Behavioural & social sciences ☐ Ecological, evolutionary & environmental sciences

For a reference copy of the document with all sections, see nature.com/documents/nr-reporting-summary-flat.pdf

Life sciences study design

All studies must disclose on these points even when the disclosure is negative.

Sample size	59 participants took part in the behavioral study and an independent sample of 64 participants took part in the MRI study. We developed a new task, that was not yet tested in the literature, therefore no explicit sample size determination was performed before collecting data from sample 1. The sample size of 59 participants is equal to in the range of typical sample sizes for studies in the field. On the basis of the effect in the behavioral sample, our target sample size was n=60 plus 15% drop outs for the MRI sample. Based on a guide for power analyses in fMRI research, it can be assumed that a sample size of N=30 is already sufficient to detect a change signal in the brain areas of interest (Mumford, 2012).
Data exclusions	Sample 1: Five participants had to be excluded from the analysis (four made to many errors in the color matching task (> 10% of trials), one person did not understand the task as communicated to the experimenter). Remaining data from 54 participants were analyzed.<br>Sample 2: Five participants had to be excluded from the analysis (two participants had a pain threshold that was too low to guarantee nociceptive processing, one person thought that they were deceived by the experimenter, one did not understand the task and made too many errors (>10% of trials) in the color matching task, one person had an incidental MR finding). A final behavioral data set of 59 subjects was analyzed from this sample. MRI data of four subjects had to be excluded from the analysis due to excessive or repeated head movement upon visual inspection, resulting in a total of 55 individual data sets for the fMRI analysis.
Replication	Behavioral results were partly replicated across the behavioral and fMRI samples. Both samples showed the same choice pattern, and the rating patterns were similar in both samples. Both samples exhibited a significant difference between the experimental conditions of controllable and unpredictable pain and between predictable and unpredictable pain. The behavioral sample showed no difference between the ratings for the controllable and predictable pain conditions, whereas we detected the effect in the fMRI sample. Based on choice behavior in the behavioral sample, we improved the matching between the experimental conditions. We explain the lack of effect in the behavioral sample by the poorer match in stimulus intensity sequences between the controllable and predictable conditions.
Randomization	The order of conditions was counterbalanced across subjects and pseudo-randomized to ensure that each condition appeared once in the first and second half of the experiment. The location and colors of the differently sized circles was randomized across trials, similar to the mapping of the colors to the different response buttons/keys. Order of thermally stimulated skin patches with was randomized for the first half of the experiment (one run of each condition) and repeated for the second part to ensure the same recovery time for each skin location. The order of stimulated skin patches was counterbalanced across subjects.

Blinding	Blinding was not possible, as the experimental process needed to be tracked outside the MR-Scanner during data collection.

Reporting for specific materials, systems and methods

We require information from authors about some types of materials, experimental systems and methods used in many studies. Here, indicate whether each material, system or method listed is relevant to your study. If you are not sure if a list item applies to your research, read the appropriate section before selecting a response.

Materials & experimental systems

n/a	Involved in the study
☒	☐ Antibodies
☒	☐ Eukaryotic cell lines
☒	☐ Palaeontology and archaeology
☒	☐ Animals and other organisms
☒	☐ Clinical data
☒	☐ Dual use research of concern
☒	☐ Plants

Methods

n/a	Involved in the study
☒	☐ ChIP-seq
☒	☐ Flow cytometry
☐	☒ MRI-based neuroimaging

Plants

Seed stocks	*Report on the source of all seed stocks or other plant material used. If applicable, state the seed stock centre and catalogue number. If plant specimens were collected from the field, describe the collection location, date and sampling procedures.*
Novel plant genotypes	*Describe the methods by which all novel plant genotypes were produced. This includes those generated by transgenic approaches, gene editing, chemical/radiation-based mutagenesis and hybridization. For transgenic lines, describe the transformation method, the number of independent lines analyzed and the generation upon which experiments were performed. For gene-edited lines, describe the editor used, the endogenous sequence targeted for editing, the targeting guide RNA sequence (if applicable) and how the editor was applied.*
Authentication	*Describe any authentication procedures for each seed stock used or novel genotype generated. Describe any experiments used to assess the effect of a mutation and, where applicable, how potential secondary effects (e.g. second site T-DNA insertions, mosiacism, off-target gene editing) were examined.*

Magnetic resonance imaging

Experimental design

Design type	Task design.
Design specifications	Each subjects underwent 6 runs of fMRI scanning, each lasted approximately 7min. FMRI signal of 90 trials with a stimulation duration of 4s per were analyzed per subject, resulting in 30 trials * 59 subjects per condition in total.
Behavioral performance measures	Button presses and response times were recorded. In the conditions, were correct performance was possible, we analyzed and excluded participants that made to many errors in the color-matching task.

Acquisition

Imaging type(s)	Anatomical, functional
Field strength	3 Tesla PRISMA (Siemens, Erlangen, Germany)
Sequence & imaging parameters	A functional MRI sequence of 50 slices (voxel size = 2.0mm3) was acquired using T2* weighted gradient echo-planar imaging (EPI; TR = 1.5s, TE = 26ms, flip angle = 60°, FOV = 224mm, multiband factor = 2, GRAPPA PAT factor = 2). A structural T1-weighted magnetization-prepared rapid acquisition gradient echo (MPRAGE) image was additionally acquired (voxel size 1.0mm3, 240 slices) before the functional runs.
Area of acquisition	50 slices, 224mm field of view, 2mm slice thickness; whole brain excluding lowest part of cerebellum.

Diffusion MRI ☐ Used ☒ Not used

Preprocessing

Preprocessing software	SPM12 in MATLAB 2020a
Normalization	Functional images were slice-timing corrected, realigned and non-linearly co-registered to the T1-weighted image. For non-

Normalization	linear co-registration the mean EPI and T1-weighted images were segmented. Nonlinear spatial normalization of the segments of the different tissue classes from the mean EPI to the T1-weighted image was performed. Finally, we computed flow fields to map the T1-weighted images to MNI space (MNI ICBM 152; 2009c Nonlinear Asymmetric). All flow fields for co-registration and normalization were computed with DARTEL.
Normalization template	MNI ICBM 152; 2009c Nonlinear Asymmetric
Noise and artifact removal	Physiological noise was modeled using the RETROICOR as implemented in the TAPAS toolbox and 24 motion regressors were added to the first-level design matrix in addition to a button press regressor of no interest.
Volume censoring	No volume censoring was applied.

Statistical modeling & inference

Model type and settings	We computed standard univariate GLMs with hemodynamic response function (HRF) convolved regressors. Onsets were defined at plateau temperature onset of the stimulus (after the rise time of the thermode to reach the temperature). Z-standardized pain ratings were added as parameteric modulators. Each boxcar regressor was defined with a duration of 4s. This resulted in one onset regressor and one modulating parametric regressor for each condition (6 regressors of interest in total). A second GLM was set up with separate pain onset regressors for each condition and added the z-scored intensity levels (not ratings) to each pain onset regressors as parametric modulators. Finally, the task period was modeled with finite-impulse response (FIR) models, starting at task onset and covering the time until stimulus onset.<br>For all models, all runs of a participant were concatenated and a session specific intercept was included. After estimating the model, the resulting beta images were warped to MNI space and smoothed with a 4mm FWHM smoothing kernel. For the second-level analyses, the normalized and smoothed beta images of the effects of interest were entered into a flexible factorial model in SPM.
Effect(s) tested	We separately analyzed pain onsets and parametric modulation of pain by ratings at the group-level with one-way ANOVAs. We computed main effects of pain onset and the condition differences in the onset model and the main effect of intensity in the model including the parametric modulators. We tested all pairwise interactions between the conditions in the model with intensity regressors. Concerning the FIR models of task, we evaluated differences between conditions at task and at the time bins relevant for pain onset by contrasting activity at the relevant time bins.

Specify type of analysis: ☒ Whole brain ☐ ROI-based ☐ Both

Statistic type for inference<br>(See Eklund et al. 2016)	voxel-wise
Correction	FWE based on random field theory as implemented by SPM12

Models & analysis

n/a	Involved in the study
☒	☐ Functional and/or effective connectivity
☒	☐ Graph analysis
☒	☐ Multivariate modeling or predictive analysis
